# A Clinical Study of Rickettsial Fever and Factors Affecting Its Outcome

**DOI:** 10.7759/cureus.63747

**Published:** 2024-07-03

**Authors:** Sachin Shivnitwar, Sunil Rajput, Ambrish Avate, Atul Narayankar, Saurabh Sujanyal, Muskaan Ahlawat

**Affiliations:** 1 Internal Medicine, Dr. D. Y. Patil Medical College, Hospital and Research Centre, Pune, IND; 2 Medicine, Shri Siddhi Vinayak Hospital, Buldhana, IND; 3 Medicine, Bidar Institute of Medical Sciences, Bidar, IND; 4 Medical Oncology, Wockhardt Hospital, Mira Bhayandar, IND; 5 Medicine, Dr. D. Y. Patil Medical College, Hospital and Research Centre, Pune, IND

**Keywords:** weil-felix test, polymerase chain reaction, scrub typhus, rickettsial infection, rickettsia

## Abstract

Introduction: Rickettsiae comprise a family of obligate intracellular short gram-negative coco-bacilli and are transmitted by insects, mites, fleas, louse, and tick vectors. Scrub typhus, north-Asian tick typhus, rickettsia pox, and boutonniere fevers are common in India and Asia. In the early phase of illness during the initial five days, all these are indistinguishable among themselves; also, they mimic any other self-limiting viral fever. Patients usually present with fever, headache, myalgia, malaise, nausea, vomiting, and anorexia. Rarely do patients present with rash, or give a history of exposure to animals or tick bite. Thus, rickettsial diseases are missed in the early phase, when they are easily treatable, due to lack of suspicion.

Aims and objectives: To study clinical features, investigations, outcomes, and factors affecting the outcome of rickettsial fever.

Materials and methods: This was an observational study conducted from December 2012 to November 2014 in a tertiary care hospital. The study population consisted of patients above the age of 13 years with a history of any one or more of the following: fever, headache, jaundice, altered sensorium, renal dysfunction, tick bite, a farmer by occupation, exposure to cattle or sheep or dog, multiorgan failure; with serological evidence of rickettsial infection by Weil-Felix test (ox-19/ox-2/ox-k ≥ 1:320) or rickettsial antibody IgM ≥ 1.1) or PCR positive. A sample size of 40 was considered for the final analysis of this study. Statistical analysis was done using inferential statistical tests such as the chi-square test and odds ratio (OR).

Result: The most common presenting symptom was fever (100%) seen in almost every patient followed by body aches (72.5%), joint pain (62.5%), and jaundice (62.5%). General examination showed icterus (37.5%), hypotension (30%), edema (22.5%), lymphadenopathy (22.5%), and pallor (15%). On the day of admission, 17 patients were found to have the Weil-Felix test positive with an OR of 0.538462 (CI = 0.151-1.917), while the Weil-Felix test done in the second week was positive in 37 patients with an OR of 5.4 (CI = 0.439-63.11). Rickettsial antibodies were positive only in three patients on the day of admission with an OR of 0.381 (CI = 0.0317-4.58), while in the second week, rickettsial antibodies were positive in 27 patients with an OR of 16.25. The rickettsial PCR test was positive in 13 patients with an OR of 1.48 (CI = 0.3857-5.722). The mortality rate was significantly high in patients presenting with breathlessness and respiratory complications like pneumonia, pulmonary edema, and acute respiratory distress syndrome. Similarly, patients presented with hypotension and required Ionotropic support had a high mortality rate.

Conclusion: While the clinical presentation of rickettsia infection is similar, the causative species and epidemiology can vary depending on the region. It is important to recognize both the typical symptoms and the epidemiology of a given region to correctly diagnose and treat these infections promptly, as they can be associated with significant morbidity and mortality. Through this study, we attempt to bring awareness about this disease which would help clinicians to suspect and start treatment at the earliest before complications set in.

## Introduction

Rickettsiae comprise a family of obligate intracellular short gram-negative cocco-bacilli. The pathogenic rickettsia is transmitted by insects, mites, fleas, louse, and tick vectors, and humans are only incidental hosts (except *Coxiella burnetii *which spreads fever by inhalational route) [1]. Rickettsial illnesses are classified according to taxonomy and diverse microbial characteristics of infectious agents, epidemiology, and clinical manifestations [2].

Epidemic typhus (louse bourn typhus, gaol typhus, classical typhus fever) was a major devastating epidemic of mankind during world war and famine as described by Hans Zinsser in his book “Rats, Lice and History." Since then, it has been seen in all parts of the world. On the other hand, the tick (Dermacentor andersoni) is a reservoir and vector for Rocky Mountain spotted fever (RMSF). They transmit pathogenic organisms both at larval and at adult stage. Once a tick is infected by *Rickettsia rickettsii*, without any harm to itself it can transmit pathogenic organisms throughout its life. Ticks can shed organisms in feces but the main route of infection is by tick bite [1]. Rickettsialpox is the mildest form of rickettsial infection. It is very similar to chickenpox, so-called vesicular or varicelli rickettsioses. Causative agent is *Rickettsia akari*; mouse mus-musculus acts as reservoir and mite Liponyssoides sanguineus acts as vector. In 1910, Conor and Bruch described a new form of spotted fever macular fever or “Boutonneuse fever.” It was associated with an inoculation eschar caused by *Rickettsia conorii*, which is transmitted to humans by the bite of the dog tick, Rhipicephalus sanguineus. This disease, later to be called Mediterranean spotted fever (MSF), was soon reported from around the whole Mediterranean basin, and also from India, the black sea, the Middle East, and South Africa [1].

Scrub typhus, or Japanese river fever, was known in Japanese folklore to be associated with the jungle mite or chigger, termed tsutsugamushi in Japanese (tsutsuga means disease, harmful, noxious and mushi means bug, insect, mite). Since 1810, the term tsutsugamushi disease has been used to describe these fevers by the Japanese. Theobald Palm in 1878 introduced this disease to Europe referring to shimamushi (island-insect) disease. The disease is transmitted by trombiculid mites when a human trespassing through the scrub of these mites is bitten by a mite larva (chigger). Chiggers feed only once throughout their life on the serum of warm-blooded animals while adult mites feed on plants. Scrub typhus pathogenic agent *Orientia tsutsugamushi* is transmitted transovarial to the progeny of mites. Rodents and birds act as reservoirs. For the disease to spread it requires a classic tetrad called a zoonotic tetrad consisting of *O. tsutsugamushi*, rats, chigger, and transitional or secondary forms of vegetation [1].

Of the above scrub typhus, north-Asian tick typhus, rickettsia pox, and boutonniere fevers are common in India and Asia [3]. All these are indistinguishable in the early phase of illness during the initial five days. Also, they mimic any other self-limiting viral fever. Patients usually present with fever, headache, myalgia, malaise, nausea, vomiting, and anorexia. Rarely do patients present with rash, or give a history of exposure to animals or tick bite. Thus, rickettsial diseases are missed in the early phase, when they are easily treatable, due to a lack of suspicion [4]. Therefore, the objective of this study was to determine the clinical features of rickettsial fevers at presentation, the social geographical background of patients, and the usage of diagnostic laboratory tests Weil-Felix test, rickettsial Ig-M test, PCR test, and factors affecting its outcome.

## Materials and methods

Ethical clearance and informed consent

The ethical clearance was obtained from the Institutional Ethics Committee prior to the start of the study. Informed consent was obtained from the participants for their willingness to participate in the study. Participation in the study was voluntary and anonymity was maintained throughout the course of the study.

Study design and data collection

This was an observational study conducted from December 2012 to November 2014 in Byramjee Jeejeebhoy Government Medical College and Sassoon Hospital, Pune, India. The study population consisted of patients above the age of 13 years with a history of any one or more of the following: fever, headache, jaundice, altered sensorium, renal dysfunction, tick bite, a farmer by occupation, exposure to cattle or sheep or dog, multiorgan failure; with serological evidence of rickettsial infection by Weil-Felix test (ox-19/ox-2/ox-k ≥ 1:320) or rickettsial antibody (IgM ≥ 1.1) or PCR positive. A sample size of 40 was considered for the final analysis of this study.

Research instrument

A detailed history with respect to their demographic features and general examination was done according to a standardized proforma with due importance to the symptomatology, residence, occupational history, clinical presentation, investigations, management, and treatment outcome of all the patients. Weil-Felix test, rickettsial IgM antibody test, and rickettsial PCR test were performed on admission. Also, the Weil-Felix test and rickettsial IgM test were performed later in the second week of illness. Liferiver machine was used for PCR and the target gene was citrate synthase (gltA). The patient was followed throughout the course of admission till discharged or death.

Data analysis

The data was entered in Excel and analyzed using IBM SPSS Statistics for Windows, Version 19 (Released 2010; IBM Corp., Armonk, New York, United States) and Epi Info 7 (Centers for Disease Control and Prevention, Atlanta, Georgia). It was summarized using percentages. Appropriate measures of association such as the OR and statistical tests such as Fischer’s exact test were used. The significance level was set at <0.05.

## Results

Demography

In this study, the most common age group affected was between 41 and 50 years. The majority of the population affected were young between 31 and 60 years (67.50%). Males (70%) are affected more commonly than females (30%). The rural population was affected more than the urban population and farmers were found to be more affected than other non-farmer occupations (students, teachers, housewives, clerks, etc.) (Table 1).

**Table 1:** Demography

Age distribution						
Age group			Number of patients	Percentage (%)		
≤20			3	7.5		
21-30			6	15.0		
31-40			6	15.0		
41-50			13	32.5		
51-60			8	20.0		
61-70			3	7.5		
71-80			1	2.5		
Total			40	100.0		
Sex distribution						
Gender		Number of patients				Percentage (%)
Male		28				70.0
Female		12				30.0
Total		40				100.0
Residence						
	Number of patients				Percentage (%)	
Rural	31				77.5	
Urban	9				22.5	
Total	40				100.0	
Occupation						
Occupation	Number of patients				Percentage (%)	
Farmer	29				72.5	
Others	11				27.5	
Total	40				100.0	

Exposure history and type of rash

In our study, most of the patients were exposed (77%) to either cattle, sheep, or dog which is the host for various vectors responsible for rickettsial infections. The rash was present in 57.5% of the total patients. Many of them presented with papular rash (40%) and the remaining had maculopapular rash.

Clinical features

The most common presenting symptom was fever (100%) seen in almost every patient. Followed by body aches (72.5%), joint pain (62.5%), jaundice (62.5%), convulsion (37.5%), breathlessness (55%), reduced urine output (27.5%), bleeding (12.5%). General examination shows icterus (37.5%), hypotension (30%), edema (22.5%), lymphadenopathy (22.5%), and pallor (15%). About 20% of patients presented with cardiac failure (S3 gallop, bilateral basal crept). Only hepatomegaly was present in 30%, while hepatosplenomegaly was present in 12.5% of patients (Table 2).

**Table 2:** Symptomatology

Symptomatology	Number of patients	Percentage (%)
Fever	40	100
Headache	21	52.5
Convulsion	15	37.5
Body ache	29	72.5
Joint pain	15	37.5
Jaundice	15	37.5
Breathlessness	22	55
Reduces urine output	11	27.5
Bleeding	5	12.5
General examination		
Sign	Number of patients	Percentage (%)
Hypotension	12	30.0
Pallor	6	15.0
Edema	9	22.5
Local lymphadenopathy	9	22.5
Icterus	15	37.5
Cardiovascular system examination		
Cardiovascular system	Number of patients	Percentage (%)
Normal	32	80.0
S3 gallop LVF	8	20.0
Total	40	100.0
Central nervous system examination		
Central nervous system	Number of patients	Percentage (%)
Normal	25	62.5
Convulsion	8	20.0
Convulsion + hemiparesis	5	12.5
Convulsion + coma	1	2.5
Hemiparesis	1	2.5
Total	40	100.0
Per-abdominal system examination		
Per-abdominal system	Number of patients	Percentage (%)
Normal	23	57.5
Hepatomegaly	12	30.0
Hepato-splenomegaly	5	12.5
Total	40	100.0

Investigation

In laboratory findings, most of the patients had hemoglobin levels >10 gm% (75%), mild anemia (22.5%), and severe anemia (2.5%). Total leukocyte count was normal in (50%), leucopenia (10%), and leucocytosis (40%). Platelet count is normal in (10%) and thrombocytopenia (90%). Deranged liver enzymes SGOT were seen in 87.5%, SGPT in 77.5%, and Bilirubin in 62.5%. Deranged creatinine was seen in 62.5% of patients. About 55% of patients present with respiratory pathology. These are ground glass opacities of 32.5%; bilateral reticulonodular shadows of 17.5% and in-homogenous opacities of 5%. USG findings include hepatomegaly in 30%, while hepatosplenomegaly in 12.5% of patients (Table 3).

**Table 3:** Laboratory investigation TLC: total leukocyte count; PLT: platelet; SGOT: serum glutamic oxaloacetic transaminase; SGPT: serum glutamic pyruvic transaminase

Parameter	Lab report		Number of patients			Percentage (%)
Hb	<7.0		1			2.5
	7.01-10.0		9			22.5
	>10.0		30			75.0
TLC	<4000		4			10.0
	4000-11000		20			50.0
	>11000		16			40.0
PLT	<50000		20			50.0
	51000-150000		16			40.0
	>150000		4			10.0
SGOT	≤40		5			12.5
	>40		35			87.5
SGPT	≤40		9			22.5
	>40		31			77.5
Bilirubin	<1		15			37.5
	1-5		16			40.0
	> 5		9			22.5
Creatinine	≤1.1		15			37.5
	>1.1		25			62.5
Chest X-ray						
Chest X-ray				Number of patients		Percentage (%)
In-homogenous opacity localized				3		7.5
Bilateral reticular shadows				7		17.5
Ground glass opacity				13		32.5
Normal				17		42.5
Total				40		100.0
USG findings						
USG findings		Number of patients			Percentage (%)	
Normal		23			57.5	
Hepatomegaly		12			30.0	
Hepato-splenomegaly		5			12.5	
Total		40			100.0	

Weil-Felix test

The Weil-Felix test was done on the day of admission and in the second week and is shown in Table no. 4. On the day of admission, 17 patients were found to have Weil-Felix test positive with OR of 0.538462 (CI = 0.151-1.917). But overall Weil-Felix test done in the second week was positive in 37 patients with an OR of 5.4 (CI = 0.439-63.11) (Table 4).

**Table 4:** Weil-Felix test

Weil-Felix test: on the day of admission	
Weil-Felix test	Total
Positive	17
Negative	23
Total	40
Weil-Felix test: results in the second week	
Weil-Felix test	Total
Positive	37
Negative	3
Total	40

A separate titer in the Weil-Felix test is shown in Table 5. Out of all titers in the Weil-Felix test, the most common titer found was OX-K, indicative of scrub typhus. OX-19 indicates the typhus fever group whereas, OX-2 indicates the spotted fever group. Therefore, in our study, most of the cases were of scrub typhus (45%) (Table 5).

**Table 5:** Separate titers in the Weil-Felix test

Weil-Felix test	Number of patients (out of total positive)	Percentage (%)
OX-19	8	20
OX-2	4	10
OX-K	18	45
OX-19 + OX-2	7	17.5
Total	37	92.5

Rickettsial antibodies

Rickettsial antibodies were done on the day of admission and repeated in the second week as shown in Table 6. Rickettsial antibodies are positive only in three patients on the day of admission with an OR of 0.381 (CI = 0.0317-4.58). When the test was performed in the second week, rickettsial antibodies were positive in 27 patients with an OR of 16.25 (Table 6).

**Table 6:** Rickettsial antibodies

Rickettsial antibodies (on the day of admission)	
Rickettsial antibody test	Total
Positive	3
Negative	37
Total	40
Rickettsial antibodies (repeated in the second week)	
Rickettsial antibody test	Total
Positive	27
Negative	13
Total	40

Rickettsial polymerase chain reaction

Rickettsial polymerase chain reaction (PCR) test was performed on the day of admission as shown in Table 7. It was positive in 13 patients with an OR of 1.48 (CI = 0.3857-5.722) (Table 7).

**Table 7:** Rickettsial PCR on the day of admission PCR: polymerase chain reaction

Rickettsial PCR	Total
Positive	13
Negative	27
Total	40

Association of outcome

Out of 40 patients, 6 patients died due to various complications. The remaining 34 patients were discharged without any disability. The association of outcome with presenting complaint, general examination, respiratory examination, per-abdominal examination, and laboratory investigation is shown in Table 8. The mortality rate was significantly high in patients presenting with breathlessness (p-value: 0.024). Similarly, patients who presented with hypotension requiring Ionotropic support had a high mortality rate (p-value: 0.006). The rest of the other signs like pallor (p-value: 0.565), edema (p-value: 0.602), lymphadenopathy (p-value: 0.602), and icterus (p-value: 0.654) are not related to poor outcomes during the study. On the other hand, patients who were presented with respiratory complications like pneumonia, pulmonary edema, and acute respiratory distress syndrome had a high mortality rate, particularly with ARDS (p-value: 0.017). Patients having hepatobiliary system involvement had a poor prognosis. Also, in laboratory tests, if a patient has deranged SGPT then that patient has a poor prognosis (p-value: 0.016).

**Table 8:** Association of outcome with presenting complaints, general examination, respiratory examination, per-abdominal examination, and laboratory investigation TLC: total leukocyte count; PLT: platelet; SGOT: serum glutamic oxaloacetic transaminase; SGPT: serum glutamic pyruvic transaminase; ARDS: acute respiratory distress syndrome

		Outcome		Total	p-value
		Survive	Death		
Presenting complaints					
Headache	Present	16	5	21	0.186
	Absent	18	1	19	
Convulsion	Present	12	3	15	0.654
	Absent	22	3	25	
Body ache	Present	24	5	29	0.999
	Absent	10	1	11	
Joint pain	Present	13	2	15	0.999
	Absent	21	4	25	
Jaundice	Present	12	3	15	0.654
	Absent	22	3	25	
Breathlessness	Present	16	6	22	0.024*
	Absent	18	0	18	
Reduces urine	Present	8	3	11	0.319
Output	Absent	26	3	29	
General examination					
Hypotension	Present	7	5	12	0.006*
	Absent	27	1	28	
Ionotropic required support	Present	7	5	12	0.006*
	Absent	27	1	28	
Pallor	Present	6	0	6	0.565
	Absent	28	6	34	
Edema	Present	7	2	9	0.602
	Absent	27	4	31	
Local lymphadenopathy	Present	7	2	9	0.602
	Absent	27	4	31	
Icterus	Present	12	3	15	0.654
	Absent	22	3	25	
Respiratory symptoms					
Consolidation	2	0	2	0.017	
Pulmonary edema	6	1	7		
ARDS	8	5	13		
Normal	18	0	18		
Total	34	6	40		
Pre-abdominal system					
Normal	22	1	23	0.038	
Hepatomegaly	9	3	12		
Hepato-splenomegaly	3	2	5		
Total	34	6	40		
Laboratory investigations					
Hb	<0.7	1	0	1	0.409
	7.01-10.0	9	0	9	
	>10.0	24	6	30	
TLC	<4000	3	1	4	0.540
	4000-11000	13	2	20	
	>11000	18	3	16	
PLT	<50000	16	4	20	0.339
	51000-150000	15	1	16	
	>150000	3	1	4	
SGOT	≤40	4	1	5	0.999
	>40	30	5	35	
SGPT	≤40	5	4	9	0.016*
	>40	29	2	31	
Bilirubin	<1	12	3	15	0.537
	1-5	15	1	16	
	>5	7	2	9	
Creatinine	≤1.1	13	2	15	1
	>1.1	21	4	25	

## Discussion

Rickettsial infections and Q fever are a common cause of acute febrile illness globally. In our study, the most common age group found was between 30-60 years, almost up to 67.5%. The mean age of affected patients is 43.6. Males (70%) are affected more than the females (30%). This data is comparable with the study done in Piau, where serum samples from 437 healthy individuals of both sexes aged from 5 to 92 years old, living in the county of Piau, state of Minas Gerais, Brazil were tested for the presence of *R. rickettsii*, *Rickettsia typhi*, *C. burnetii* phase 1 and phase 2, *Bartonella henselae*, *Bartonella quintana*, and *Ehrlichia chaffeensis* IgG and IgM antibodies. Age over 40 years old was associated with more seropositivity for *R. rickettsii*, *C. burnetii* phase 1, *B. henselae*, *B. quintana*, and *E. chaffeensis* [5].

Many rickettsioses are accompanied by a maculopapular, vesicular, or petechial rash or sometimes an eschar at the site of the tick or mite bite. In our study, rash was found in 57.5% of patients. Specifically, Papular non-blanching rash is seen in 40% of patients, while maculopapular rash is seen in 17.5% of patients. The rash was mostly located on the trunk (80%) and abdomen (39%). It was also present in upper limb (82%) and lower limb (74%). Weerakoon et al. did a descriptive study of skin manifestation in rickettsial diseases and found that out of the eight body regions considered, the majority of patients had the skin rash in their arms and forearms (n = 108, 81%) and legs (n = 90, 67%) while other areas involved were palms (74, 55%), soles (75, 56%), anterior trunk (61, 46%), thighs and gluteal region (53, 40%), head and neck (45, 34%), and posterior trunk (44, 33%). The type of cutaneous lesion they had were macula-papular in 132 (98.5%), macular in 1 patient, and papular in another one. Eight patients (6%) of the group had fern-leaf type skin necrosis and the rest had erythematous rash [6].

Clinical presentations vary with the causative agent and patient; however, common symptoms that typically develop within 1-2 weeks of infection include fever, headache, malaise, rash, nausea, or vomiting. In our study, the most common presenting symptom is fever (100%) seen in almost every patient. Followed by body aches (72.5%), joint pain (62.5%), jaundice (62.5%), convulsion (37.5%), breathlessness (55%), headache (52.5%), reduced urine output (27.5%), bleeding (12.5%). The study done by Liu et al. in northern China over 12 years from 1995 to 2006 who studied 480 cases of scrub typhus showed that the common clinical symptoms of scrub typhus were fever (100.0% of confirmed cases), rash (90.4%), eschar (88.5%), and regional lymphadenopathy (60.6%). Headache (100.0%), myalgia and prostration (100.0%), loss of appetite (82.9%), chills (67.3%), abdominal pain (48.8%), erythematous flushes (48.8%), nausea/vomiting (43.8%), retro-orbital pain (17.3%), and flank tenderness (10.0%) were observed, respectively [7]. Also, general examination in our study shows icterus (37.5%), hypotension (30%), edema (22.5%), lymphadenopathy (22.5%), and pallor (15%). Per abdominal findings were hepatomegaly present in 30%, while hepatosplenomegaly was present in 12.5% of patients. This is consistent with the study by Dr. Rathi on rickettsial fever in central India they found that rash is present in 83% of patients, edema (92%), hepatomegaly (99%), and lymphadenopathy (41%) [8].

Laboratory investigations play a huge role in diagnosing infectious diseases. In our study, 2.5% were found to have severe anemia, 40% had leucocytosis and the majority had deranged liver enzymes. A study done by Dass et al. also showed that hyponatremia (66.7%), elevated liver enzymes (58.3%), and thrombocytopenia (26%) were the other significant laboratory findings [9]. On the day of admission, 17 patients in our study were found to have Weil-Felix test positive whereas 37 patients were positive when the test was performed in the second week. Rickettsial antibodies were positive in only three patients on the day of admission but 27 patients came out to be positive when the test was performed in the second week. The rickettsial PCR test was positive in 13 patients with an OR of 1.48 (CI = 0.3857-5.722) on the day of admission. The study carried out by Mahajan showed that the Weil-Felix test is insensitive in diagnosing scrub typhus; it may come negative when carried out during the first week of illness. It is thus advisable to carry out the Weil-Felix test in the second week or later. It would be advisable to start treating rickettsial diseases at presentation, and then confirm the diagnosis with the help of the Weil-Felix test later in the second week. A study of rising titers as mentioned by Mahajan may also prove helpful [10]. In our study, we have not studied the rising titer but the finding of repeat titers shows a rising titer in the second week. Our study also suggests the same thing that treating the patient on the basis of clinical suspicion and confirmatory tests can be done in the second week like the Weil-Felix test and rickettsial antibody.

Mortality was compared with different parameters. Our study shows mortality rate was significantly high in patients presenting with breathlessness (p-value: 0.024), hypotension (p-value: 0.006), patients requiring Ionotropic support (p-value: 0.006), respiratory complication (p-value: 0.017), hepatobiliary system involvement, deranged SGPT (p-value: 0.016). Wang et al. studied acute respiratory distress syndrome in scrub typhus. This retrospective study showed that 11.1% of the 72 scrub typhus patients had ARDS complications. The mortality rate for the eight scrub typhus patients with ARDS was 25% (two of eight) [11]. In our study for rickettsial illness, out of 13 acute respiratory distress syndrome patients five (38.5%) were dead. A Pubmed, Medline, Cochrane Library's search for literature in the past 40 years was carried out by Dr. N. Rather and Dr. A. N. Rathi revealed that rickettsial infections are reemerging and prevalent throughout the world. In India, cases have been reported from Tamil Nadu, Maharashtra, Karnataka, Kerala, Jammu and Kashmir, Uttaranchal, Himachal Pradesh, Assam, West Bengal, and Rajasthan. The low index of suspicion, nonspecific signs and symptoms, and unavailability of diagnostic tests readily lead to late diagnosis and mortality even though the treatment is simple [12].

## Conclusions

Rickettsia is a group of vector-borne organisms that cause acute febrile illnesses throughout the world. While the clinical presentation of rickettsia infection is similar, the causative species and epidemiology can vary depending on the region. It is important to recognize both the typical symptoms and the epidemiology of a given region to correctly diagnose and treat these infections promptly, as they can be associated with significant morbidity and mortality. Through this study, we attempt to bring awareness about this disease which would help clinicians to suspect and start treatment at the earliest before complications set in.
